# Editorial: Highlights in oral infections and microbes 2021/2

**DOI:** 10.3389/froh.2022.1040789

**Published:** 2022-11-02

**Authors:** Georgios N. Belibasakis, Oleh Andrukhov, Robert P. Allaker

**Affiliations:** ^1^ Division of Oral Diseases, Department of Dental Medicine, Karolinska Institutet, Stockholm, Sweden; ^2^ Competence Center for Periodontal Research, University Clinic of Dentistry, Medical University of Vienna, Vienna, Austria; ^3^ Centre for Oral Immunobiology & Regenerative Medicine, Institute of Dentistry, Queen Mary University of London, London, United Kingdom

**Keywords:** oral infections and microbes, saliva, periodontal disease, epithelial barrier, irrigation, endodontics, laboratory diagnostics, antimicrobials

**Editorial on the Research Topic** Highlights in oral infections and microbes 2021/2

Upon launching Frontiers in Oral Health, the inaugural editorial of the section Oral Infections and Microbes addressed the contemporary challenges of the field (1). A series of Research Topics commissioned thereafter were summarized in the respective editorials (2, 3). The Highlights in Oral Infections and Microbes 2021/2 Research Topic collection, detailed in this Editorial, was announced to showcase articles of contemporary importance in the field of oral microbiology and infection biology. A set of two articles of clinical and one of translational relevance were compiled and discussed further here. The first one brings into spotlight the value and potencies of laboratory microbiology in clinical diagnosis and treatment of oral diseases Claesson et al. (2022), whereas the second article Tonini et al. (2022) and its associated corrigendum Tonini et al. (2022) discuss the effectiveness of various irrigating solutions and activation systems for root canal disinfection, raising the necessity for developing of standardized effective clinical protocols. The third article employed an *in vitro* model to evaluate the effects of saliva on oral epithelial barrier function Roy et al. (2022).

Conventional laboratory diagnostics can deliver supportive microbiological evidence to facilitate clinical decision-making Claesson et al. (2022). Cultivation and molecular methods can be readily available in clinical laboratory environments. Periodontal, endodontic and odontogenic infections are identified as pathologies whose diagnosis or treatment could be assisted by clinical microbiology laboratory analysis for the benefit of the patient. Administration of antimicrobial agents, backed by prior microbiological analysis, can yield more predictable treatment outcomes in refractory or early occurring forms of periodontitis. Confirmation of a sterile root canal by means of a culture-negative sample during endodontic treatment may ensure the longevity of the treatment outcome. Antimicrobial susceptibility testing on samples obtained from odontogenic abscesses may guide the selection of the appropriate antimicrobial, in order to prevent further spread of the infection.

Root canal system disinfection is an essential part of endodontic therapy, aiming to eliminate bacterial biofilm and prevent subsequent infection (4). Traditionally used mechanical biofilm debridement does not always result in satisfactory bacteria elimination because of strong biofilm attachment to the surface, hiding bacteria in dentinal tubules, and the complex anatomy of the root canal system. A further possibility for root canal disinfection is the irrigation with disinfection solution, such as sodium hypochlorite or chlorhexidine. This procedure improves the elimination of bacteria but is not always sufficiently compelling. The effectiveness of the irrigation procedure could be further enhanced by using the activation system, which promotes the movement of the irrigating agent through the root canal system and allows better penetration within the biofilm. Tonini et al. analyzed the effectiveness of various irrigating solutions and activation systems in root canal disinfection using a systematic review tool Tonini et al. (2022). The authors searched through different databases and found seven randomized controlled trials (RCT), which met the inclusion criteria and were included in the review. In four RCTs, only the irrigants in different combinations were used, whereas, in the other three RCTs, various activation techniques were additionally applied. Based on the data extraction and analysis, the authors concluded that using the activation technique is essential to root canal disinfection. Further, the application of sodium hypochlorite is indispensable in endodontic therapy. Finally, the authors emphasize the necessity of developing standardized clinical protocols for effective bacterial elimination from the root canal.

There is a general lack of knowledge as regards the effect of saliva from subjects with periodontitis on the integrity of the epithelial barrier and the inflammatory response. The effects of unstimulated saliva from healthy and periodontitis subjects were compared with respect to these parameters using *in vitro* models of the oral epithelium Roy et al. (2022). Saliva samples were analysed using an immunological multiplex assay to assess the levels of cytokines and metalloproteinases (MMPs) relevant to periodontitis. The impact of saliva on epithelial barrier integrity was assessed by monitoring transepithelial electrical resistance (TER) in an oral epithelium / keratinocyte model. Oral epithelial cells were also treated with saliva from both groups to determine their ability to induce the secretion of interleukin 6 (IL-6) and IL-8. Saliva from the periodontitis subjects contained significantly higher concentrations of MMP-8, MMP-9, IL-8, and chemokine ligand 1 compared healthy subjects. Saliva from both groups increased TER and induced IL-6 and IL-8 secretion to a similar extent. It is known that saliva from periodontitis subjects contains higher levels of pro-inflammatory mediators, MMPs, and bacteria-derived toxic products. It would be of interest to assess a wider range of such salivary biomarkers, including those of microbial origin, and determine any possible synergistic or inhibitory effects between these molecules.
